# Space Flight-Promoted Insulin Resistance as a Possible Disruptor of Wound Healing

**DOI:** 10.3389/fbioe.2022.868999

**Published:** 2022-05-13

**Authors:** F. Strollo, S. Gentile, A. M. V. Pipicelli, A. Mambro, M. Monici, P. Magni

**Affiliations:** ^1^ Endocrinology and Metabolism Unit, IRCCS San Raffaele Pisana, Rome, Italy; ^2^ Department of Internal Medicine, Campania University “Luigi Vanvitelli”, Naples, Italy and Nefrocenter Research Network, Naples, Italy; ^3^ Nephrology, Dialysis and Transplant Unit, Medical and Surgical Sciences Department, “A. Gemelli” Sacred Heart Catholic University, Rome, Italy; ^4^ Anesthesiology and Intensive Care Unit, Pertini General Hospital, Rome, Italy; ^5^ Department of Experimental and Clinical Biomedical Sciences “Mario Serio”, ASA Campus Joint Laboratory, ASA Res. Div, University of Florence, Florence, Italy; ^6^ Department of Pharmacological and Biomolecular Sciences, Università Degli Studi di Milano, Milan, Italy; ^7^ IRCCS Multimedica Hospital, Sesto San Giovanni, Milan, Italy

**Keywords:** insulin resistance, wound healing, microgravity, spaceflight, rehabilitation, diabetes complications

## Abstract

During space flight, especially when prolonged, exposure to microgravity results in a number of pathophysiological changes such as bone loss, muscle atrophy, cardiovascular and metabolic changes and impaired wound healing, among others. Interestingly, chronic low-grade inflammation and insulin resistance appear to be pivotal events linking many of them. Interestingly, real and experimental microgravity is also associated to altered wound repair, a process that is becoming increasingly important in view of prolonged space flights. The association of insulin resistance and wound healing impairment may be hypothesized from some dysmetabolic conditions, like the metabolic syndrome, type 2 diabetes mellitus and abdominal/visceral obesity, where derangement of glucose and lipid metabolism, greater low-grade inflammation, altered adipokine secretion and adipocyte dysfunction converge to produce systemic effects that also negatively involve wound healing. Indeed, wound healing impairment after traumatic events and surgery in space remains a relevant concern for space agencies. Further studies are required to clarify the molecular connection between insulin resistance and wound healing during space flight, addressing the ability of physical, endocrine/metabolic, and pharmacological countermeasures, as well as nutritional strategies to prevent long-term detrimental effects on tissue repair linked to insulin resistance. Based on these considerations, this paper discusses the pathophysiological links between microgravity-associated insulin resistance and impaired wound healing.

## Introduction

Humans engaged in space flight show a number of physiological changes due to a set of known (microgravity, confinement, isolation, space radiation) as well as still unknown stressors related to this specific environmental condition (White and Averner 2001). The opportunity of prolonged or even very prolonged space flights, like long-term stay at the International Space Station (ISS) or the planned travel to the Moon, Mars and possibly to other planets, represents a major health challenge for astronauts, since exposure to the above-mentioned factors may result in relevant and even permanent pathological changes, such as bone loss, muscle atrophy, dysregulation of the immune system, cardiac and metabolic alterations and impaired wound healing (White and Averner 2001; Frippiat et al., 2016; Grimm et al., 2016; Riwaldt et al., 2017), to mention just some of them. Among these stressors, microgravity has been extensively studied by means of in-flight as well as on-Earth experiments, such as the 6-degrees head down tilt (HDT) (Grimm 2021). Microgravity is known to promote a series of cardio-metabolic changes, including fluid shift, impaired glucose and lipid metabolism, increased oxidative stress and pro-inflammatory cytokine release and chronic low-grade inflammation through muscle unloading and other causes. Interestingly, a central pathophysiological event linking most of these processes is insulin resistance at specific organs, like the liver, the skeletal muscles and the adipose tissue, but also the blood vessels and the skin and epithelia (Tobin et al., 2002; Bergouignan et al., 2011).

In the context of space flight, the relevance of correct wound repair is increasingly important, since, in addition to unwanted injuries, some surgical procedures may also be implemented during prolonged space missions (Drudi et al., 2012). However, several studies indicate that microgravity can induce an impairment of processes specifically related to wound repair (Delp 2008), for example by altering the functionality of cell populations involved in such events (Cialdai et al., 2020). Thus, wound healing impairment after traumatic events and surgery in space remains a relevant concern for space agencies.

According to knowledge obtained from on-Earth biomedicine, it has been clearly observed that chronic wounds, with defective repair processes, are a common complication in patients with type 2 diabetes mellitus (T2DM) and the related insulin resistance, and often lead to amputation. Such non-healing wounds are characterized by a persistent inflammatory state promoted by pro-inflammatory macrophages, pro-inflammatory cytokines and proteases (Salazar et al., 2016).

Based on these considerations and within the specific context of astronaut health during prolonged space flight and exposure to microgravity, the occurrence of insulin resistance may thus contribute to the observed impairment of wound healing (Bacci and Bani 2022). This paper discusses the evidence and knowledge gaps regarding this pathophysiological association. To this aim, the PubMed and Excerpta Medica Database (Embase) were searched from inception until April 2022. Used search terms, with a combination of MeSH terms if applicable in each database, included: (space flight), (microgravity), (insulin resistance), (wound), (healing).

## Insulin Resistance and Metabolic Syndrome Pathophysiology and Assessment

**FIGURE 1:**
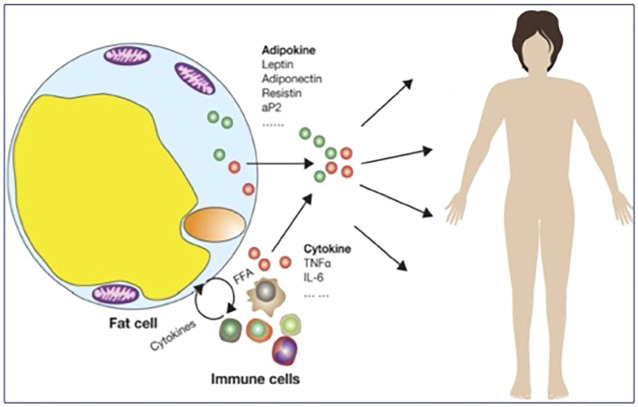
Mechanisms underlying chronic low-grade inflammation after excess/ectopic/dysfunctional adipose tissue accumulation (adapted from (Cao 2014)).

Insulin resistance is the pathophysiological hallmark of the metabolic syndrome (MS), which is currently a global epidemic, accounting for some 15% or more of the overall population worldwide, and is tightly linked to increased obesity prevalence and chronic low-grade inflammation (Saklayen 2018; Neeland et al., 2019; Ross et al., 2020). The MS is defined as a cluster of cardio-metabolic clinical conditions (increased waist circumference, arterial hypertension, hyperglycemia, reduced HDL cholesterol and increased triglyceride), which results in increased individual risk for cardiovascular morbidity and mortality (Alberti et al., 2009). In this context, a pivotal role in the pathophysiology of the MS and the related increased cardiovascular risk is played by the expanded and dysfunctional visceral adipose tissue compartment (Neeland et al., 2019; Ross et al., 2020). Indeed, far from having a mere storage function, adipose tissue can be referred to as a diffuse endocrine organ (Ahima 2006), secreting several intertwined signals which contribute to appetite and energy regulation, immunological and metabolic balance, and wellbeing. These molecules include the adipokines leptin and adiponectin, which modulate a relevant set of physiological functions along with other signals from the gut (ghrelin) and other organs (Magni et al., 2000; Bertoli et al., 2006; Dozio et al., 2009; Magni et al., 2009), and whose changes are associated with important alterations of cardio-metabolic functions (Norata et al., 2007) and bone metabolism in patients (Magni et al., 2010), as well as under microgravity conditions. When growing well above its normal volume or outside its natural boundaries into the abdominal space and other sites, adipose depots soak the liver, the pancreas, and other organs with fatty acids and cause local metabolic changes leading to excess oxygen demands. As a consequence, adipose tissue attracts macrophages bound to get rid of damaged cells and releasing high amounts of pro-inflammatory cytokines, in addition to altered adipokine amounts released by dysfunctional adipocytes (Magni et al., 2005; Cao 2014) (Figure 1).

**FIGURE 2:**
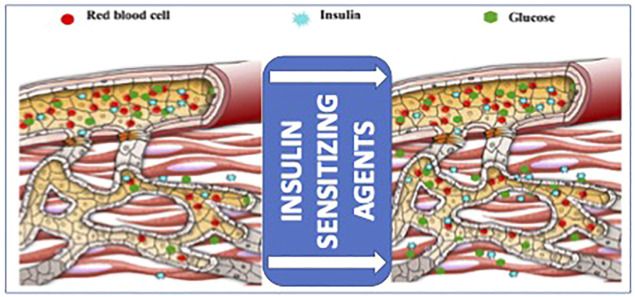
Schematic diagram of the effects of insulin sensitizing agents, including adiponectin, on microvascular recruitment and insulin delivery in the muscle, providing an at least partial explanation of the negative impact of insulin resistance on anabolic signal-related wound healing (adapted from (Zhao et al., 2014)).

These changes tend to progress over time since hypertrophic fat depots expand spontaneously through various self-maintaining mechanisms related to chronic low-grade inflammation, associated polarization of macrophages to the Th2 phenotype (Figure 2) and triggered by adipokines and other factors (Cao 2014), including insulin resistance and muscle dysfunction (Dyck et al., 2006), that are also characteristic of the aging process, and, therefore, can be defined “inflammaging” ((Franceschi and Campisi 2014; Franceschi et al., 2018; Fülöp et al., 2019).

Moreover, nephropathy is frequently observed within the frame of an ill-regulated aging process due to metabolic aberrations negatively affecting acid-base balance, calcium, glucose, and lipid metabolism (Samaras and Campbell 2005). Insulin resistance is characterized by impaired insulin activity, which is initially compensated for by higher circulating insulin levels. Individuals with severe insulin resistance report: 1) altered glucose metabolism including type 2 diabetes mellitus (T2DM) or paradoxical late post-prandial hypoglycemia; 2) acanthosis nigricans, velvety hyperpigmentation of axillary and flexural skin; and 3) hyperandrogenism in women (Savage et al., 2007).

Different approaches have been proposed to identify and monitor insulin resistance in humans. Fasting insulin levels only reflect a specific time point and are influenced by dynamically intertwined metabolic components, including overnight hepatic glucose production and its effects on insulin output, hepatic insulin extraction, spontaneous pancreatic insulin secretion spikes, and the balance between kidney gluconeogenesis and insulin excretion rate (Laakso 1993). Thus, fasting insulin levels are quite variable within the same individual and unreliable, when considered alone, for diagnostic approach.

The “Homeostasis Model Assessment (HOMA)-1” (Radziuk 2014) and “HOMA-2” (Levy et al., 1998) index calculations can be easily adapted to large-scale studies (Demir et al., 2020), thanks to their straightforward mathematic approach. Hyper-insulinemic euglycemic clamp (HIEGC) is the reference method for quantifying insulin resistance, but it is a complex, time-consuming, and expensive method, thus less likely to be utilized in specific experiments, like those designed for space flight studies (Johns et al., 2012). In the future, promising results are expected from metabolomics (Milburn and Lawton 2013). Recently, an indirect non-invasive method has been developed based on pulse wave velocity analysis, taking advantage of impaired vascular elasticity as a function of inflammaging, but yet still awaits final validation as for insulin resistance when applied to clinical research (Petrasek et al., 2015). The potential usefulness of measurement of the circulating levels of leptin and adiponectin may also be considered, as the former is known to have pro-inflammatory effects, while the latter acts as an insulin sensitizer, a good marker of insulin sensitivity, counteracts apoptosis and reduces inflammation in various cell types (Lihn et al., 2005; Yadav et al., 2013; Caselli 2014; Achari and Jain 2017). Therefore, the leptin/adiponectin ratio has also become a sensitive marker of insulin resistance by combining two intertwined signals into a single parameter (Norata et al., 2007; Frühbeck et al., 2018; Frühbeck et al., 2019).

## Microgravity and its Effects on Human Health: Focus on Glucose Metabolism and Insulin Resistance

Space flight is characterized by unloading-dependent muscle atrophy and impaired immune response as well as different degrees of metabolic dysregulation, which, if persisting long enough, as expected during interplanetary missions with extended duration, might result in increased risk of cardiovascular and metabolic diseases (Lane and Smith 1999; Strollo 1999; Demontis et al., 2017; Strollo et al., 2018a). More specifically, space flight research, ground-based studies (both bed rest and dry immersion), and experimental studies in animal models and on pancreatic islets of Langerhans support the observation that prolonged space flight and experimental exposure to microgravity result in peripheral insulin resistance at different sites (liver, skeletal muscle, adipose tissue and others) (Tobin et al., 2002; Linossier et al., 2017; Wang et al., 2019; Downs et al., 2020; Strollo and Vernikos 2021). Under these environmental conditions, insulin resistance specifically occurs also in other insulin-sensitive tissues, such as the blood vessels (Hughson et al., 2016). Moreover, slight dehydration and inadvertently occurring traumas can cause skin lesions that might suffer healing problems during such missions based on the aforementioned mechanisms. To elaborate a holistic view of the long-term effects of space flight, to understand the resulting health risks, and, possibly, to unravel the mechanisms leading from temporary functional alterations to the onset and progression of chronic diseases, it is needful to shed light on the concurrent outcomes of the multiple alterations concomitantly occurring at those three levels. Indeed, altered metabolism, sarcopenia, and immune deficiency represent models of Earth-bound age-related chronic diseases having oxidative stress and consequent chronic low-grade inflammation as the common underlying mechanisms (Mancuso 2016; Nishida and Otsu 2017).

To this regard, a significant issue is to dissect the confounding effects of multiple space-related stressors, which are direct contributing causes of pathophysiological adaptation processes in the space environment. Stressful tasks and diet changes, confinement-related artificial light, isolation, and reduced motor activity might worsen the effects of weightlessness and radiation by further contributing to muscle and immune system deconditioning and to altered energy metabolism and endocrine balance (Strollo et al., 2014; Strollo et al., 2018a; Strollo et al., 2018b). Indeed, several studies conducted during manned space missions highlighted endocrine alterations in the crew (Strollo 2000; Macho et al., 2001) potentially linked to weightlessness and stress factors including confinement, isolation, demanding tasks, and circadian rhythm disturbances. For instance, rodent research showed that psychosocial stressors and altered gravity could affect immune function and inflammatory mediators known to interact with metabolism (Gaignier et al., 2018). However, we currently know that insulin sensitivity changes occur both in space (Tobin et al., 2002; Hughson et al., 2016) and in Earth-bound simulated microgravity by bed rest esperiments (Downs et al., 2020) and long-term-flight-simulating isolation experiments (Strollo et al., 2014; Strollo et al., 2018b). Indeed, it is well established that prolonged (60–70 days) head-down bed rest studies are associated with significant insulin resistance in healthy volunteers, which is worsened by inactivity and is only partially improved by physical exercise (Downs et al., 2020). A similar finding has also been observed in male subjects undergoing a 3-days dry immersion experiment (Linossier et al., 2017).

Environmental stress, associated to confinement, which is a typical feature of space flight, is another relevant factor disrupting insulin sensitivity, as observed in crew members participating to the Mars-105 and Mars-500 on-ground simulation mission (Strollo et al., 2014; Strollo et al., 2018b).

Moreover, the Mars-500 human volunteers showed significantly lower plasma levels of total and high-molecular weight adiponectin, especially in the first 120 days of mission, which is also supporting the impact of environmental stress upon metabolic adaptations and significant glucose metabolism changes, independently of microgravity contribution (Strollo et al., 2018b).

Vitamin D3 deficiency is also known to negatively affect insulin sensitivity and astronauts are at risk for vitamin D3 deficiency, due to chronic exposure to artificial light conditions. Presumably due to that, vitamin D supplementation has proved beneficial in wound healing of people with T2DM and foot ulcers (Urbaniak and Reid 2016). Another factor potentially contributing to insulin resistance during space flight is the altered gut microbiota environment (Razzaghi et al., 2017; Turroni et al., 2017). Interestingly, urbanization is strongly related to changes (including reduced biodiversity) in stool bacteria composition through intrinsic isolation from the surrounding “bacteria-rich” country. Similarly, due to artificial lighting, closed environment, and confinement, space flight might be considered as an extreme “urban-like” potentially diabetogenic condition (Tasnim et al., 2017; Parajuli et al., 2018; Wang et al., 2018). Indeed, some space-related studies reported a negative impact on gut microbiota composition in experimental models and humans (Ritchie et al., 2015; Cervantes and Hong 2016; Harada et al., 2016; Mahnert et al., 2021; Siddiqui et al., 2021).

Taken together, the available data suggest that strong evidence is present about spaceflight impact on insulin resistance, although future studies are still needed to identify specific exercise, nutritional and nutraceutical countermeasures to mitigate such deleterious pathophysiological event.

### Metabolic Alterations and Impaired Wound Healing

Obesity, and specifically abdominal/visceral obesity, is a condition of greater insulin resistance and chronic inflammation (Bashir et al., 2016), as well as markedly impaired cutaneous wound healing. This concept also applies to T2DM, a catabolic disease typically characterized by a severe insulin-resistant condition leading to hyperglycemia and a global metabolic derangement. In most cases, T2DM represents the natural evolution of a long-standing, often obesity-driven insulin resistance state slowly exceeding functional pancreatic reserve and associated with insulin receptor or post-receptor abnormalities. On the other hand, type 1 DM (T1DM) is not only the consequence of severe beta-cell deficiency, but is also burdened, over time, by some degree of insulin resistance due to insulin overtreatment-related receptor downregulation, advanced glycation endproducts (AGE) accumulation, and more (Kaul et al., 2015). Then, through several pathophysiological mechanisms, including insulin resistance, both DM types undergo macro- and micro-vascular complications over time. These complications are tightly linked to higher cardiovascular risk, renal damage, eventually resulting in chronic kidney disease (Ndisang et al., 2017; Vladu et al., 2017; Wolosowicz et al., 2020). In any case, apart from their extreme post-ischemic expressions, i.e. the diabetic foot and severe leg ulcerations, wound-healing problems often represent an underestimated complication in this context. When the wound is ischemic, the resolution of neutrophils and macrophages appears to be significantly delayed. Excess pro-inflammatory cells release reactive oxygen species (ROS), cytokines, and metalloproteinases, thus generating and maintaining a pro-inflammatory microenvironment that further increases tissue damage, extracellular matrix (ECM) degradation and denaturation of growth factors (Mizuno et al., 2004). An abnormal inflammatory response is also a major feature and the leading cause of impaired diabetic wound healing. Dysfunctional repair is even more apparent in the presence of high levels of local advanced glycation end-products (AGEs), which impair ECM deposition by inducing fibroblast apoptosis and dysfunction of ECM production. The imbalance of deposition and degradation of ECM affects keratinocyte and endothelial cell function, eventually leading to impaired re-epithelialization and angiogenesis (Yang et al., 2016).

Increasing evidence has accumulated on insulin ability to stimulate cell migration and wound recovery, and insulin administration has been proposed to overcome the adverse effects of insulin resistance on wound healing on the ground (Yang et al., 2016). Physical exercise typically decreases insulin resistance and speeds up cutaneous wound healing in aged mice (Goodson and Hunt 1979). The interplay between insulin-mediated glucose supply and wound healing mechanisms is schematically shown in Figure 2.

All these pathogenetic events deserve careful consideration in light of upcoming long-duration interplanetary travels (Demontis et al., 2017) and, even more, of Moon colonization missions, potentially placing astronauts at risk for higher cardiovascular disease risk, in addition to altered wound healing and other dysfunctions.

## Effects of Microgravity on Wound Healing: Potential Role of Increased Insulin Resistance

An important health problem of astronauts in space is skin deterioration and this may specifically impact on the wound repair process, due to exposure to microgravity (Gössl et al., 2010; Choi et al., 2021; Bacci and Bani 2022), radiation (Hellweg and Baumstark-Khan 2007; Hu et al., 2020) and other factors. Wound healing is a complex series of events including partially overlapping phases (inflammation, tissue formation, and tissue remodeling) (Eming et al., 2014). This process is based upon a series of cell populations playing specific roles at different stages of the repair process (Pasparakis et al., 2014), which should follow a precise sequence of events. Mechanical factors, among different biochemical and physiological factors, play a significant role in wound healing, and include skin tension, mechanical forces at the wound margins, mechanical stress produced by stitches (after suture) and wound contraction due to myofibroblasts (Darby et al., 2014). The coordinated modulation of the expression of a large number of genes in the involved cells is clearly important for the success of the whole repair process. Moreover, a relevant role is played by the apoptotic process in all phases of wound healing, balancing cell growth and elimination of cells that are not necessary anymore (Riwaldt et al., 2017). Several studies show that microgravity can impair repair processes (Delp 2008), altering the behavior of some cell populations involved in wound repair (Morbidelli et al., 2005; Riwaldt et al., 2017). Experimental studies showed alterations in the behavior of cutaneous cell lineages under microgravity, especially regarding the apoptosis process in wound healing (Riwaldt et al., 2021). Moreover, altered modulation of haemostasis is also possible under microgravity condition, since some *in vivo* and *in vitro* studies show that microgravity affects the number and function (i.e., production of platelet-derived growth factor) of platelets, which contributes to impair wound healing (Farahani and DiPietro 2008; Locatelli et al., 2021). Another critical event in wound repair is physiological angiogenesis which occurs in the granulation tissue to allow supply of oxygen and nutrient supply and removal of waste products. Thus, understanding the alterations of angiogenesis during real and experimental microgravity conditions will allow to understand this component of wound repair alterations during space flight (Morbidelli et al., 2021). Currently, no specific studies have addressed the issue of the selected impact of insulin resistance on (experimental) wound healing during real or simulated space flight. In this regards, the Earth-based paradigm of patients with T2DM, with important microvascular complications and relevant wound repair impairment, may suggest at least in part what are the consequences of marked/prolonged insulin resistance on the complex process of wound healing (Fu et al., 2021; Rodríguez-Rodríguez et al., 2022), and suggest some potential experimental approaches to begin to disentangle this pathophysiological problem.

## Conclusion and Future Developments

Among the multiple health issues that are observed during a long-term stay in microgravity conditions, like prolonged space flight, to achieve appropriate wound healing represents an important goal, as this repair process could often be impaired by different factors, including insulin resistance. This goal may be relevant for both minor injuries and even for some (possibly limited) surgical procedures that may need to be conducted during longer space flights. Thus, some countermeasures should be at least envisioned to manage this issue, possibly following a personalized approach, in a context of precision medicine. Starting from the individual health profiling, it is conceivable, for example, to propose an accurate monitoring of individual insulin resistance and chronic low-grade inflammation by wearable devices, in order to identify the astronauts at greater risk. Moreover, monitoring of the visceral adipose compartment, even by simple waist circumference measurement, may be another way to assess personal susceptibility (Ross et al., 2020). Additionally, a nutritional profile that mitigates insulin resistance may be crucial and could be based upon food with lower glycemic load and possibly rich in antiinflammatory and antioxidant components. Such approach may then be conducted with a plant-based diet, like, for example, the Mediterranean diet (Schwingshackl et al., 2015). Interestingly, engineering progress has recently been done to develop strategies to produce nutritious and palatable food directly on spacecrafts involved in interplanetary missions (Douglas et al., 2020). In particular, a suitable way to produce edible vegetables on spaceship and carbon dioxide absorption and oxygen production to maintain the air circulation system is currently under development (Jiang et al., 2020). Such a choice could also ensure an excellent solution against environment-related oxidative stress (Kyriacou et al., 2017). A further strategy may also include the validation and use of specific food supplements with nutraceutical properties (curcumin, berberine, etc.) and, where necessary, selected drugs targeted to reduce insulin resistance (Rochlani et al., 2017).

The development of effective countermeasures able to mitigate the association of insulin resistance and impaired wound healing during prolonged space flight thus deserves further studies. In this context, our group has been suggesting investigating this topic since a long time and now has finally been selected for a space experiment dealing with it. Next studies will need to combine nutrition, supplements and pharmacological approaches also with any possible physical, endocrine and metabolic countermeasures to prevent long-term detrimental effects on tissue repair potentially related to any long-lasting, unopposed insulin resistant state.
